# An Update and Perspectives on Mitochondrial Membrane Protein-Associated Neurodegeneration and *C19orf12* Research

**DOI:** 10.3390/brainsci15080777

**Published:** 2025-07-22

**Authors:** Barbara Gnutti, Arcangela Iuso, Chloé Angelini, Dario Finazzi

**Affiliations:** 1 Section of Biotechnology, Department of Molecular and Translational Medicine, University of Brescia, 25123 Brescia, Italy; barbara.gnutti@unibs.it; 2 Institute of Human Genetics, School of Medicine and Health, Technical University of Munich, 81675 Munich, Germany; arcangela.iuso@helmholtz-munich.de; 3 Institute of Neurogenomics, Helmholtz Zentrum München, 85764 Munich, Germany; 4 Service de Génétique Médicale, Hôpital Pellegrin, CHU Bordeaux, 33000 Bordeaux, France; chloe.angelini@chu-bordeaux.fr; 5 Centre de Référence Maladies Rares «Neurogénétique», Service de Génétique Médicale, CHU Bordeaux, 33000 Bordeaux, France; 6 Centre National de la Recherche Scientifique, Institut de Neurosciences Cognitives et Intégratives d’Aquitaine, UMR 5287, NRGen Team, University of Bordeaux, 33076 Bordeaux, France

**Keywords:** Mitochondrial Membrane Protein-Associated Neurodegeneration (MPAN), *C19orf12*, Neurodegeneration with Brain Iron Accumulation (NBIA), movement disorders, rare hereditary diseases

## Abstract

Mitochondrial Membrane Protein-Associated Neurodegeneration is a rare monogenic form of neurodegeneration characterized by iron accumulation in the brain. It is due to variants in the orphan gene *C19orf12*. Since its definition in 2011, many scientific groups have investigated the clinical features and molecular underpinnings of the disorder. In this review, we summarize the main points of progress in this field, trying to highlight the issues that need further attention and efforts to speed up the diagnostic path, improve the existing treatment options, and define targeted therapies.

## 1. Neurodegeneration with Brain Iron Accumulation Disorders

Neurodegeneration with Brain Iron Accumulation (NBIA) disorders are a set of rare, heterogeneous neurologic diseases sharing the eponymous sign of iron deposits in the brain [1]. The discovery of the first genetic determinant for a subtype of these movement disorders, namely variants in the *pantothenate kinase 2* (*PANK2*) gene [2], together with the increased access to deep sequencing technologies, paved the way for the search and identification of several genetic associations. At the moment, variants in 10 different genes are linked with subtypes of NBIA, while more genetic variants in six additional genes await further validation (Table 1) [3,4]. Pantothenate Kinase Associated Neurodegeneration (PKAN), Beta-propeller Protein-associated Neurodegeneration, Mitochondrial Membrane Protein-associated Neurodegeneration (MPAN), and Phospholipase A2 group 6-associated Neurodegeneration (PLAN) are the most common forms, representing more than 90% of all affected individuals. These disorders present with a wide spectrum of clinical symptoms including dystonia, parkinsonism, chorea, pyramidal involvement, speech disorders, cognitive decline, and ocular anomalies [4,5]. Treatment remains largely symptomatic, even though, for some of the disorders, relevant clinical trials are undergoing and could lead to the identification of disease-modifying therapies [4,6]. This review focuses on MPAN, briefly outlining the clinical features of the disease and summarizing the results obtained so far for the definition of *C19orf12* gene function and the understanding of its connection with neurodegeneration. We searched the PubMed database of the NIH-National Library of Medicine (PubMed) using *C19orf12* as a key term, retrieving 114 items. We discarded 10 of them, not related to MPAN or NBIA, and analyzed the remaining 104.

## 2. MPAN Disorder

### 2.1. Clinical Phenotype

MPAN is a very rare inherited neurologic disorder with an estimated prevalence of one in 1,000,000 [7] and a lifetime risk of 0.01 per 100,000 [8]. The disorder is included in the NBIA category, representing about 5–10% of all NBIA patients. Generally, it is due to biallelic, loss-of-function (LOF) variants (autosomal recessive pattern, AR-MPAN) in the *C19orf12* gene, even though patients with autosomal dominant transmission (AD-MPAN) or de novo heterozygous variants have been described [9]. Interestingly, these different genetic forms present overlapping clinical phenotypes. The onset occurs in childhood (juvenile form) or in early adulthood (adult form); the progression is slow; and most patients survive well into adulthood. However, some adult patients with rapid progression have been described [10,11]. The disorder manifests with a broad phenotypical spectrum (Table 2) characterized by the variable presence of spasticity, psychiatric changes, vision disturbances, dystonia, motor axonal neuropathy, autonomic dysfunctions, and cognitive decline [12]. Common symptoms at the onset are walking and gait changes such as toe walking, spastic gait, and frequent falls; spasticity, predominantly in the lower limbs; psychiatric abnormalities including anxiety, inattention, and hyperactivity; and vision impairment associated with optic atrophy [13]. Noteworthy, MPAN clinical features may show a great variability among patients, and the symptomatology may mimic other disorders, such as Behr syndrome [14], pallido-pyramidal syndrome [15], amyotrophic lateral sclerosis [16,17,18], or hereditary spastic paraplegia [19,20,21,22,23]. The MRI finding of iron accumulation in the basal ganglia is relevant to orient the diagnosis correctly. Progressive dystonia involving hands and feet or more generalized, dysarthria, dysphagia, cognitive decline, and parkinsonism are common clinical features, with severe dementia, spasticity, hyper-reflexia, dystonia, and parkinsonism being most prominent in the end stage of the disorder [7]. A multisystem involvement can appear, with the presence of heart, muscle, gastro-intestinal, and urogenital tract problems [7,24]. People with advanced disease often present with marked weight loss and bowel and/or bladder incontinence, and may require the use of a wheelchair. Death typically occurs due to complications such as aspiration pneumonia. A recent study investigated clinical phenotype and disease progression by cross-sectional evaluation of eighty-five MPAN patients [13]. The authors calculated a median diagnostic delay of five years, followed by a gradual worsening of symptoms over approximately fifteen years. After this period, about half of the patients are wheelchair dependent. Subsequently, a plateau phase occurs, marked by a stabilization of the symptoms. Gender, genotype, age of onset, and ethnicity did not correlate with the progression rate. In contrast with a previous report that associated older age at onset, higher incidence of parkinsonism and optic atrophy with the homozygosity for one of the most common MPAN variants (c.-2C>T; nomenclature of variants refers to transcript NM_031448.6) [25], the study did not identify patient subpopulations. This could be due to the low number of patients carrying such a variant in the case study. Importantly, these authors suggested the need for a MPAN-specific rating approach in order to improve the description of the disease’s natural history.

### 2.2. MRI and Spectroscopy Findings

The detection of signs indicative of iron accumulation in the basal ganglia is an essential step for the diagnosis of MPAN (or more generally of NBIA), then confirmed by the genetic workup. Typical MRI features observed on T2/T2*-weighted images or susceptibility-weighted imaging sequences are bilaterally symmetric hypointensities of the globus pallidus (GP) and the substantia nigra (SN) [11,26]. A typical radiological sign of MPAN is the presence of a band of high signal intensity in the GP, called the internal medullary lamina. This sign, although not pathognomonic, is highly specific to MPAN and can be mistaken for the “eye of the tiger” sign [27], a typical finding in PKAN. Furthermore, some MPAN patients with a true “eye of the tiger” feature have been described [28,29,30]. Other peculiar findings have been described, such as the “comb like” appearance of the substantia nigra in three Indian affected individuals [31]. In older patients with long disease duration and/or late onset, signs of iron deposits were observed also in the caudate nucleus and putamen [32,33] together with cerebellar and cortical atrophy. White matter hyperintensities, localized mainly in the periventricular regions, may be seen in some MPAN patients at different stages of the disease [26]. Neuroimaging features are similar in dominant and recessive MPAN patients [34]. These features were confirmed in a quantitative susceptibility mapping with ultrahigh field MRI study, which found significantly higher susceptibility not only in the GP and SN but also in the caudate nucleus of four MPAN patients [35], suggesting the involvement of this structure in MPAN. Surprisingly, the magnetic susceptibility in the caudate nucleus and putamen was also increased in some heterozygous carriers of *C19orf12* variants (for AR-MPAN), in the absence of overt clinical symptoms. Further investigation is necessary to understand the relevance of the data in the process of iron accumulation and the involvement of selected brain regions in the pathogenesis of MPAN.

### 2.3. Neuropathology

The first examination of a brain from a MPAN patient reported the presence of iron deposits, enriched in the GP and SN [28]. Neuronal depletion, α-synuclein-positive Lewy bodies, Lewy neurites, and axonal spheroids were evident in several brain regions, together with neuronal inclusions containing hyperphosphorylated tau, particularly abundant in the hippocampus. Loss of myelin was seen in the pyramidal tracts of the spinal cord and optic nerve. Similar results were found in brain biopsies or other brain samples from affected individuals [11,32], including patients with AD-MPAN [9,36].

### 2.4. Genetics

**Figure 1:**
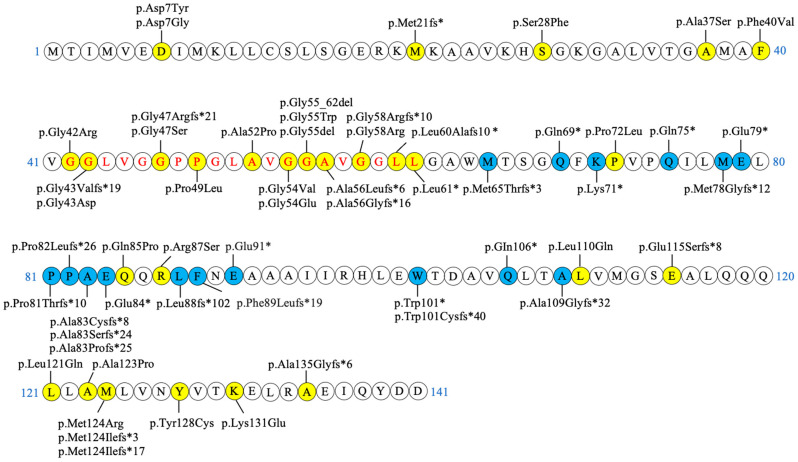
Schematic representation of MPAN-related variants on the *C19orf12* isoform 2 (NM_031448.6). Variants highlighted in yellow are biallelic, light blue ones are monoallelic. The amino acids predicted to form the transmembrane domain (MEMSAT SVM prediction tool, see below) are indicated in red.

**Figure 2:**
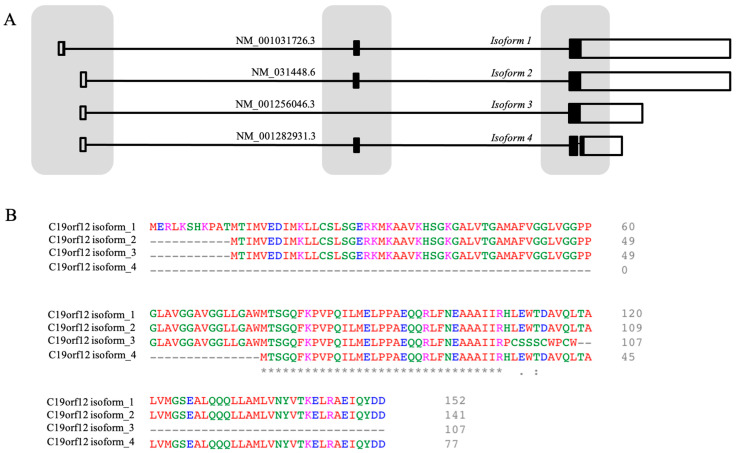
Predicted transcripts and protein isoforms for human *C19orf12* gene. (**A**) Schematic representation of the alternative *C19orf12* transcripts. Black rectangles are coding regions; white rectangles are non-coding regions. Lines are introns. (**B**) Alignment of the predicted protein isoforms. Residues are colored according to their physicochemical properties. Asterisks (*) indicate positions with a single, fully conserved residue; colons (:) indicate conservation between groups with strongly similar properties; and periods (.) indicate conservation between groups with weakly similar properties.

About two hundred MPAN individuals and fifty different variants in the *C19orf12* gene have been described in the literature (Table 3 and Table 4 and Figure 1). Most patients are homozygous or compound heterozygous for deleterious missense/nonsense variants (AR-MPAN) (Table 3), but nucleotide changes affecting splice sites [37,38,39] and complete exon deletions have been described [40,41]. Two variants are largely represented in the MPAN population. The c.171_181del (p.Gly58Argfs*10) variant has an allelic frequency of approximately 30% and was first identified in a cohort of NBIA individuals from Poland, where haplotype analysis indicated a common founder [28]. The c.-2C>T variant, on the other hand, is more frequent among patients of Turkish descent [25], accounting for about 25% of all documented *C19orf12* variants. Interestingly, this latter variant affects the untranslated 5′-region of the transcripts encoding both the canonical *C19orf12* isoform (NM_031448.6, producing a 141-amino acid protein) and alternative transcripts (NM_001256046.3 and NM_001282931.3) predicted to encode shorter proteins. Additionally, it impacts the coding region of transcript NM_001031726.3, which was previously considered the canonical isoform and encodes a protein of 152 AAs (see below and Figure 2). Patients with the c.-2C>T homozygous variants appear to have a later onset (mean age of onset 25.7 years) [25]. Several studies identified a significant number of patients carrying single, heterozygous variants and showing a clinical phenotype largely overlapping with that of autosomal recessive individuals [36,42,43,44,45,46,47]. These variants (Table 4) are indels or nonsense changes that induce premature termination of the coding sequence and localize on the last exon of the gene. They are associated with a dominant pattern of inheritance or are de novo variants [36], as confirmed by the description of heterozygous MPAN patients that are offspring of mosaic individuals [9]. While LOF variants are clearly associated with the recessive pattern of disease inheritance, less straightforward is the interpretation of the autosomal-dominant transmission of the disorder. An interesting hypothesis suggests that truncated proteins, resulting from variants in the last exon, exert a dominant-negative effect by assembling with the wild-type protein from the second allele, thereby interfering with its functionality. This would explain the large overlap in the clinical features of AR- and AD-MPAN, both linked to a LOF of the protein [36]. Noteworthy, there is no definitive evidence that *C19orf12* forms dimers or multimers. An alternative explanation comes from the observation that different mRNA and protein isoforms are predicted for the *C19orf12* gene (see below). The variants linked to AD-MPAN are potentially damaging for all the putative protein isoforms, thereby leading to a condition of haploinsufficiency, even at the heterozygous state. On the contrary, LOF variants linked to AR-MPAN and located upstream of codon seventy-five in the canonical isoform (NM_031448.6) would not affect the coding sequence of the shortest isoforms. The persistence of these proteins could guarantee some residual functionality, and a second variant is necessary to generate a clinical phenotype [45]. Also, in this case, the expression of the shorter isoforms is hypothetical at the moment. More in-depth comprehension of the biology of the *C19orf12* gene will help in elucidating this aspect.

### 2.5. Therapy

Treatment is generally symptomatic. Baclofen, trihexyphenidyl, intramuscular botulinum toxin, and bilateral pallidal deep brain stimulation are used to mitigate dystonia and spasticity. Levodopa can be used for parkinsonism with variable response. A few patients have been treated with deferiprone, an iron-chelating agent that permeates the blood–brain barrier. In two patients, the treatment was associated with stabilization of the symptomatology [62,64]; in the others, there was no benefit, even in the presence of reduction of iron content in the SN, or the drug had to be discontinued because of side effects or worsening of the clinical features [62]. Surprisingly, the treatment had the opposite effect even when applied to two sisters carrying the same mutation [62]. It is important to emphasize that deferiprone was administered as compassionate therapy, not within the framework of a clinical trial.

## 3. *C19orf12* Gene and Protein

### 3.1. The Gene

The human *C19orf12* gene (ID 83636) localizes on the long arm of chromosome 19 (19q12). The release of v1.3 of the Matched Annotation from the NCBI and EMBL-EBI project annotates several transcripts derived from alternative splicing (Figure 2). At least three different proteins are predicted, with 141 (Uniprot Q9NSK7-4, hereafter isoform two), 107 (Uniprot Q9NSK7-3, isoform three), and 77 (Uniprot Q9NSK7-2, isoform four) AAs (Figure 2). Previous NCBI and EMBL-EBI annotation releases associated a protein of 152 AAs (Uniprot Q9NSK7-1, isoform one) with the transcript variants NM_001031726.3. Interestingly, isoform one, two, and four differ exclusively in their length, with isoform two and four lacking the first eleven and seventy-five AAs of isoform one, respectively. Isoform three lacks the first eleven and the last thirty-four (119–152) AAs of isoform one, and the last ten residues (98–107) of its COOH terminal portion are completely different from those found in other isoforms. We know very little about the real existence and biological relevance of these different protein isoforms. Since the initial discovery of the association between *C19orf12* gene and MPAN, isoform one has been considered the canonical form [28]; this interpretation was supported by the identification of a common variant (c.-2C>T) in MPAN patients, affecting the coding sequence of isoform one (eleventh codon) and the 5′ untranslated region of the other isoforms. Recently, the Western blotting analysis of *C19orf12* protein content in fibroblasts from patients and controls and in different human tissues, evidenced the presence of two bands, most probably corresponding to the 152- and 141-AA-long proteins. The latter was clearly more abundant. No other band with a lower molecular weight was detected [65]. This is in line with the most recent releases of gene databases, which identify the isoform of 141 AAs as the canonical form not only in humans, but also in most species. The presence of the isoforms of 107 and 77 residues or other lengths is predicted by the NCBI and Ensembl databases. Verifying the real existence of the different protein isoforms and their specific distribution in different tissues and organs could be useful to understand *C19orf12* biology and the functional impact of variants found in MPAN patients. As mentioned above, it could allow explaining the dual modality of inheritance observed in patients and the specific vulnerability of the brain areas more heavily affected by the neurodegenerative process.

### 3.2. The Structure of the C19orf12 Protein

**Figure 3:**
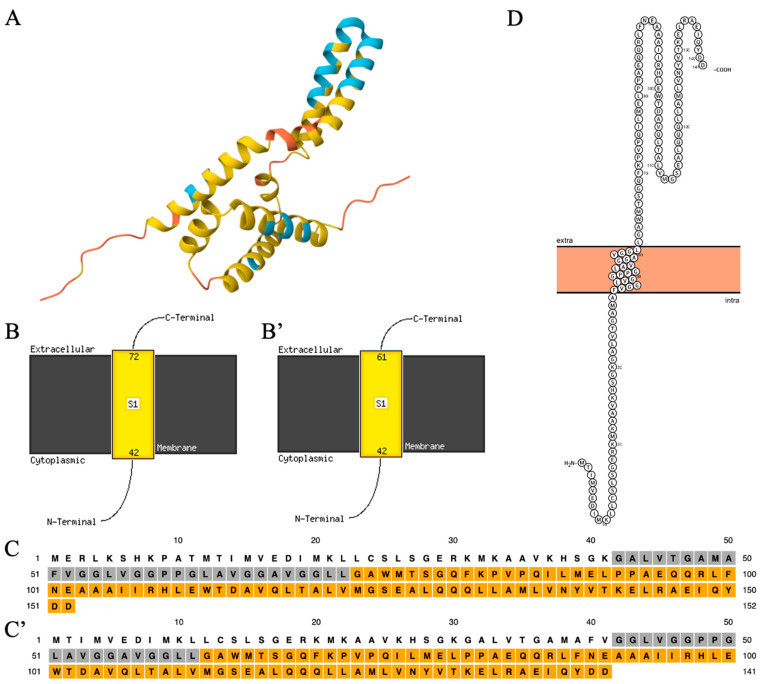
In silico modeling of *C19orf12* protein structure and topology. (**A**) Alphafold prediction of *C19orf12* protein structure. The colours represent the confidence of the model: light blue = high; yellow = low; orange = very low. (**B**,**B’**) Proposed topologies for *C19orf12* protein (152- and 141-AA-long isoforms) by the MEMSAT-SVM web tool. (**C**,**C’**) PSIPRED prediction of *C19orf12* (152- and 141-AA-long isoforms) protein domains. Grey shading = hydrophobic, transmembrane domain; orange shading = luminal/extracellular domain; no shading = cytoplasmic domain. (**D**) Proposed topology for the *C19orf12* protein (141-AA-long isoforms) by the Protter web tool.

Little work has been performed so far to characterize the *C19orf12* protein. Venco and colleagues [66] carried out the in silico prediction of the secondary structure and topology of the protein (152-AA-long isoform). The PSIPRED-MEMSAT3 [67] analysis predicted a transmembrane region (residues 41–80) containing two parallel alpha-helices and positioning the soluble N- and C-termini of the protein in the cytosol. The length of the predicted hydrophobic domain and the presence of single or paired proline residues within the region in all known species further support the possibility that this domain forms a hydrophobic hairpin. While this prediction appears to be similar to the alphafold prediction [68,69] (https://alphafold.com) (Figure 3A), the analysis with the newest release of the same web-based tool (MEMSAT SVM) gives different results. The transmembrane domain is modeled as a single-pass alpha helix (residues 42–72), positioning the soluble extremities of the protein at the opposite sites of the membrane, with the N-terminus inside the organelle and the C-terminus outside (Figure 3B,C). Similar results are obtained for the 141-AA-long isoform, including the prediction by the PROTTER web tool (https://wlab.ethz.ch/protter/) (accessed on 8 July 2025) (Figure 3B’,C’,D). Interestingly, the transmembrane region contains several glycine residues, many of which were found mutated in MPAN patients, arranged to form glycine zipper motifs, generally GXXXCXXXG. This pattern is frequently present in membrane protein sequences [70] and represents a common motif, where the glycines act as a driving force for right-handed packing against a neighbouring helix. It is thus possible that the glycine zipper motifs of *C19orf12* determine the interaction between the two transmembrane helices of the protein, when the alphafold model is correct. Alternatively, it could act as a guiding force for homo- or hetero-multimerization.

### 3.3. The Intracellular Localization of C19orf12 Protein

**Figure 4:**
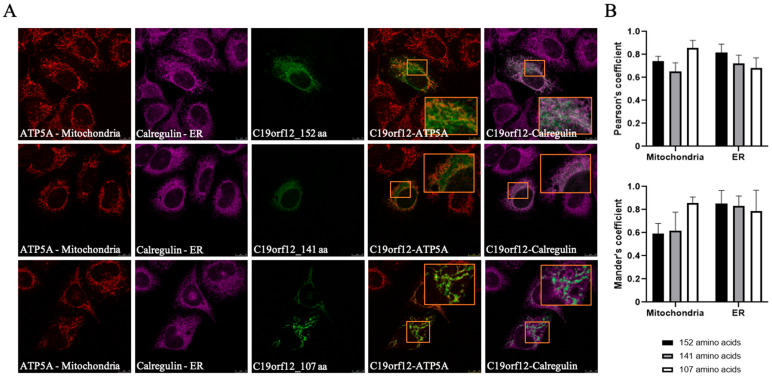
Intracellular localization of the *C19orf12* protein isoforms. (**A**) Representative confocal images of HeLa cells overexpressing *C19orf12* isoforms of 152, 141, and 107 amino acids. Mitochondria (red) were labeled with an ATP5A antibody, the ER (magenta) with an anti-calreticulin antibody, and *C19orf12* (green) was visualized via its -tGFP tag. Insets show magnified views of merged green-red and green-magenta channels. Scale bar: 10 µm. (**B**) Colocalization analysis using Pearson’s correlation coefficient and Mander’s overlap coefficient (MOP), representing the proportion of tGFP signal overlapping with mitochondria or ER (n = 10 cells). Data are mean ± SD.

Several groups investigated the cellular distribution of *C19orf12* protein. Most data come from immunofluorescence studies performed in cells overexpressing the long isoform of 152 AAs, usually as an epitope-tagged protein [19,28,66]; recently, Mori et al. have investigated the localization of the endogenous protein in SK-MEL-28 human skin melanoma cells [71]. The results show that the protein resides in the endoplasmic reticulum (ER), mitochondria, and the cytosol. Cellular fractionation and in vitro import approaches [28,66,71] indicated the association with the outer membrane of mitochondria and the localization in membrane-associated mitochondria (MAM), regions of tight contact between ER and mitochondria. Interestingly, recent data have documented the presence of *C19orf12* protein at the surface of lipid droplets in human adipocytes [72]. So far, no one has investigated the cellular distribution of other predicted *C19orf12* protein isoforms. We cloned the different predicted *C19orf12* isoforms with either the Green Fluorescent Protein (GFP) or Halo-tag at the C-terminus and expressed them in HeLa cells (File S1: Materials and Methods in the Supplementary Information). The images show that the isoforms with 152, 141, and 107 AAs have a similar distribution in the cells: GFP fluorescence clearly stained the ER and mitochondria, as documented by the extensive overlap with the organelle markers (Figure 4). Interestingly, the distribution of the 107 AAs isoform appeared to be more restricted to the mitochondria. Similar results were obtained with the Halo-tagged proteins. The overexpression of the 77-AA-long protein appeared to be toxic to the cells, since GFP fluorescence was repeatedly detected in very few dying cells. We can conclude that the 141-AAs isoform has an intracellular localization identical to that of the longer one. In the absence of the confirmation of the physiological expression of shorter isoforms, we can draw limited information from their overexpression. The conserved ER-mitochondria distribution of the 107-AAs form, which lacks the last COOH-terminal AAs, suggests that this region is not fundamental for the intracellular trafficking of the protein. Higher level of colocalization with the mitochondrial marker allows us to infer possible changes in the interaction profile or strength of the protein, which may affect its targeting to the ER. The data show that *C19orf12* can be either membrane-associated or in the cytosol. When cells overexpressing *C19orf12* were exposed to an oxidative insult, the protein changed its distribution and became more cytosolic, suggesting a loose and potentially regulated membrane tethering [66]. This phenomenon is apparently in contrast with the structural model predicting a transmembrane domain with two alpha helices for the *C19orf12* protein and should be investigated more carefully.

### 3.4. The Function of C19orf12

#### 3.4.1. *C19orf12* and Lipid Metabolism

The exact function of the protein is still largely undefined, even though we have evidence for connections with several cellular processes and activities. It increases the data point to a role of *C19orf12* in lipid metabolism; the gene is ubiquitously expressed in human tissues, but highest levels are found in the adipose tissue [28,73]. In whole blood, the expression of the gene was co-regulated with that of genes involved in lipid-related biochemical pathways, such as the fatty acid (FA) synthesis and the branched-chain AA degradation [28]. Furthermore, the *C19orf12* protein level increased constantly during the course of adipocyte differentiation in vitro [28]. More recently, a study investigating changes in protein distribution and level during adipogenesis in vitro [72] identified *C19orf12* as a lipid droplet protein. Data from co-immunoprecipitation and silencing experiments revealed a possible role as a lipid droplet–mitochondrial connector, participating in the control of lipid turnover and FA utilization in mitochondria. Interestingly, the deletion of *CG11671* (*Nazo*) and *CG3740* genes, the two fly orthologs of human *C19orf12*, caused significant changes in the expression level of genes involved in lipid metabolism [74]; furthermore, the knockout of *Nazo* led to a sharp decrease in lipid droplets in enterocytes of mutant flies, associated with a significant reduction in the whole-body triglyceride content, locomotor deficits and shorter lifespan. These phenotypes were rescued by the overexpression of Nazo or Perilipin-2, a lipid droplet protein, which maintains lipid droplets by antagonizing the lipase Brummer. Similar results were derived from the silencing of Brummer itself. The authors concluded that *Nazo* LOF led to diminished Perilipin-2 and increased lipolysis due to excess activation of the lipase Brummer. Even though the results are partially in conflict with those generated in human adipocytes, where *C19orf12* silencing caused increased lipid droplets, decreased basal lipolysis, and increased stimulated lipolysis [72], they let us infer a relevant connection between *C19orf12* and lipid movement/transport inside the cells. We do not know if this is also the case in neural cells, and a recent investigation of the lipidomic profile in MPAN patients’ fibroblasts showed a modest change in the content of some neutral lipids and phospholipids, especially in the galactose-containing medium [51]. Nonetheless, a detailed study performed in a *Drosophila* model of PLAN supports the relevance of the *C19orf12*/lipid metabolism connection also for the neurons [71]. *iPLA2*-VIA/*PLA2G6* gene encodes an enzyme that hydrolyzes the sn-2 ester bonds of phospholipids, generating free FA and lysophospholipids [75]. This process has a major role in the remodeling pathway (Lands’ cycle), in which the cycle of phospholipid deacylation and reacylation modifies the FA composition to generate a mature membrane [76]. The deletion of *PLA2G6* in *Drosophila* led to the appearance of signs of neurodegeneration with synaptic transmission impairments and locomotor defects due to changes in the lipid composition of membranes. A significant shortening of phospholipid acyl chains led to ER stress and perturbation of neuronal activity. Interestingly, the overexpression of *dPLA2G6,* as well as of *dC19orf12* (*CG3740*) or *hC19orf12*, rescued the aberrant biochemical, structural, and behavioral phenotypes. Even though the exact molecular mechanism of the rescue was not identified, the authors suggested that the increase in the number of MAM associated with the overexpression of *C19orf12* could play a significant role. Altogether, we can speculate that *C19orf12* can participate in the movement and distribution of lipids between different organelles in the cells, contributing to the control of membrane fluidity, curvature, structure, and dynamics on one side and the usage of lipids for energetic purposes on the other. It could be of interest to investigate whether *C19orf12* may act as a docking site for proteins that permit the bridge-like transport of lipids between membranes [77].

#### 3.4.2. *C19orf12* and Autophagy

The first indication of a possible connection between *C19orf12* and the autophagy process came from the analysis of starved HeLa cells overexpressing *C19orf12*-EGFP [66], which showed a higher number of puncta with colocalization of *C19orf12* and LC3, an increase in the LC3II/LC3I ratio, together with a lower level of p62. These changes were less evident or absent in cells exposed to oxidative stress, causing the cytosolic redistribution of the protein, or overexpressing mutant p.Gly47Ser (c.139G>A) and p.Gln85Pro (c.254A>C) forms of *C19orf12*, suggesting a localization-dependent involvement of *C19orf12* in the control of autophagy. An initial confirmation for such a role came from the analysis of fibroblasts derived from MPAN patients: a subtle defect (reduced LC3II/LC3I ratio) in basal autophagy was detected upon bafilomycin exposure. The defect was exacerbated when mitophagy was induced, exposing cells to the protonophore carbonyl cyanide *m*-chlorophenyl hydrazine. Interestingly, the overexpression of *C19orf12* not only restored but even increased the number of LC3 fluorescent puncta in the cells, suggesting a contribution of the protein to early events of autophagosome formation [65]. A similar reduction in the LC3II/LC3I ratio was documented in fibroblasts from patients with AD-MPAN [9], while the recent work of Wydrych et al. [51] has evidenced defective LC3I to LC3II conversion in fibroblasts grown in conditions promoting mitochondrial respiration. At the moment, we lack any mechanistic insight into the possible connection between the *C19orf12* protein and the autophagy flux. Since the autophagy flux is not blocked, but slightly dampened, we propose an indirect role of the protein in this process. The ER provides the lipids/membranes for the initial formation and growth of the autophagosomes, including the phosphatydilethanolamine (PE) required for LC3 lipidation; considering the potential role of *C19orf12* in lipid transport among different organelles, it is possible that the lack of *C19orf12* leads to changes in ER membrane composition (lower level of long chain FA [71] or PE [65]) and/or fluidity and negatively affects the dynamic process of autophagosomes budding and maturation.

#### 3.4.3. *C19orf12* and Mitochondria/Oxidative Stress/Iron Homeostasis

The deletion of the *C19orf12* gene in the M17 neuroblastoma cell line led to changes in mitochondrial morphology associated with impaired mitochondrial respiration. The cells showed higher levels of cytosolic iron, higher susceptibility to the treatment with ferroptosis inducers such as erastin, increased reactive oxygen species (ROS) with signs of lipid peroxidation [78]. The mitochondrial phenotype was largely confirmed in SH-SY5Y neuroblastoma cells carrying a heterozygous dominant variant in the *C19orf12* gene [47], in two fibroblast lines, and the brain parenchyma from MPAN [78]. These results are in contrast with those obtained by Zanuttigh et al. [65], who did not find significant changes either in mitochondrial morphology and bioenergetics and in ROS content in several fibroblast lines from MPAN patients, but in line with a more recent study that confirmed the presence of shorter mitochondria, the derangement of mitochondrial respiration with reduced levels of OXPHOS complexes III and V, and lower cellular capacity to oxidize fatty acids and tricarboxylic acid cycle substrates [51]. The level of ROS was slightly increased in basal conditions, and even more upon metabolic reprogramming of the cells by incubation in medium with galactose instead of glucose, even though signs of increased oxidative damage to proteins or lipids were absent. Changes in iron and ferritin content were more evident in fibroblasts from AD-MPAN patients, which had a modest response to the treatment with ferric ammonium iron [8], suggesting an impairment in the maintenance of iron homeostasis. Iron accumulation was not found in a *Drosophila* model of MPAN [79] even though *Nazo* and *Nazo*/*CG3740* double mutants exhibited higher sensitivity to oxidative stress [73]. The variability of these results could be related to differences in experimental conditions, combined, in the case of fibroblasts studies, with differences in variant types and genetic backgrounds. Notably, in Zanuttigh et al.’s work [65], fibroblasts from carriers of AR-MPAN were used as the control group to ensure a genetic background more closely resembling that of the patients. Altogether, the changes in iron homeostasis, mitochondrial functionality, and response to oxidative stress appear to be indirect manifestations of a yet-to-be-defined function of *C19orf12*.

### 3.5. Genotype–Phenotype Correlation

Very few variants have been investigated for the biological impact so far. As described above, two distinct categories can be set apart for the mechanism of action and pattern of inheritance: most variants are inherited in a recessive manner and hence interpreted as LOF variants. Indeed, the Western blotting analysis of lysates from fibroblasts from AR-MPAN patients revealed the complete absence of *C19orf12* protein. Epitope-tagged mutant forms of *C19orf12* overexpressed in mammalian cells may display a change in the intracellular distribution, with a predominant cytosolic localization (p.Gly47Ser, p.Ala52Pro, p.Gly55del, p.Gly58Arg) [19,28,66], or a vesicular pattern with a partial co-localization with the mitochondrial and ER compartments (p.Gln85Pro). When compared with the wild-type protein, the mutant ones are less efficient in changing their intracellular distribution upon exposure to an oxidative stimulus [66]. It is interesting to observe that many LOF variants affect glycines in the putative transmembrane domain (p.Gly42Arg, p.Gly54Glu, p.Gly58Arg) and are expected to disrupt the localization in membrane compartments. An interesting variant is the (c.-2C>T); it affects the eleventh AA of the longer isoform and non-coding regions of the other forms. Interestingly, it appears to be associated with a later onset of the disease [20,25]. Considering the recent results from the analysis of patients’ fibroblasts showing higher levels of the 141-AA-long protein, this variant indicates the necessity for a more detailed investigation of the biological role and tissue-specific expression of the putative *C19orf12* isoforms. Another set of variants is found at the heterozygous state; in most individuals, they are de novo ones, but in some cases, inherited in a dominant manner or even as mosaic variants [9]. They should exert a dominant negative effect, blocking the activity of the protein encoded by the wild-type allele. Western blottings performed in patients’ fibroblasts (p.Glu91*, p.Lys71*) [9] or KI neuroblastoma lines (p.Pro81Trefs*9) [47] show a strong reduction or even the complete absence of the protein, suggesting an effect of the variants on the total amount of *C19orf12* protein. It seems that these dominant variants are associated with more severe biological perturbations, including iron accumulation and mitochondrial deficiency, even though the clinical phenotype is usually not different from that due to recessive variants. Altogether, we miss a detailed analysis of the impact of each variant on *C19orf12* protein structure and function, which is most probably bound to the limited information we have about the biology of the protein itself.

## 4. Animal Models of *C19orf12* Deficiency

The consequences of *C19orf12* deficiency were investigated in two animal models, i.e., *Drosophila melanogaster* and *Danio rerio* (zebrafish). *Drosophila* has two orthologs of the human *C19orf12* gene, *CG3740* and *CG11671* (*Nazo*). While the silencing of each single gene had no effects in flies, the downregulation or heterozygous deletion of both genes resulted in a significant shortening of the lifespan, limited climbing performance, and altered responses in the bang sensitivity assay. The brain of twenty-eight-days-old double RNAi flies showed a higher number of vacuoles, indicative of significant tissue loss [79]. Reduction in longevity and perturbed climbing activity were also documented in another *Nazo* mutant fly [74], which showed severe perturbation in triglyceride homeostasis in the enterocytes, as described above. Interestingly, overexpression of *Nazo*, but not of *CG3470*, resulted in a significant decrease in viral replication in S2 *Drosophila* cells, and the opposite was obtained by its silencing. This led to the identification of Nazo as an effector of the STING-IKKβ activation pathway controlling infections by picorna-like viruses [80]. The mechanism involved in such activity as well as its relevance in mammalian cells are completely unknown. Interestingly, proteins that control the mammalian innate immune response to viruses (STING and MAV) share some commonalities with *C19orf12*: they are localized in the ER, mitochondria and MAMs, and have connections with lipid metabolism and lipid post-translational modification of proteins [81,82,83]. Further investigations of these features could lead to relevant information about *C19orf12* biological activity. The role of *c19orf12* was also investigated in zebrafish embryos. The fish has four different co-orthologs of the human gene. One of them, *c19orf12a*, is expressed at a higher level during embryonal development and particularly in the CNS [84]. Its downregulation by injection of a specific morpholino perturbed brain morphology and led to smaller head and eyes, reduced yolk extension, and a tilted and thinner tail. These signs were associated with changes in the distribution and intensity of expression of selected neural markers, perturbed musculature formation, and defective locomotor behaviour. Stable KO lines are under investigation to extend the study to adult fish.

## 5. Conclusions

The association of variants in the *C19orf12* gene with MPAN dates back to 2011 [28]. Since then, many manuscripts have reported the clinical features of several patients contributing to the definition of standardized diagnostic criteria and the basis for natural history description [7,11,13,85]. Advancements in understanding the pathogenic mechanisms have been more limited. We do not know the biological function of the *C19orf12* protein yet. Most data obtained so far describe phenotypes associated with the presence of mutant alleles in cells from patients, in cells overexpressing mutant forms of the protein, or in cells and animals with deletion of the gene. They indicate that defective *C19orf12* affects lipid metabolism, mitochondrial function, autophagy flux, and intracellular iron homeostasis. The molecular connection linking the protein with these biological processes is poorly understood, and this is a major limitation in the search for targeted therapeutic strategies for the disease. Recent data documenting the capacity of the protein to largely suppress the phenotype associated with the deletion of dPLA2G6 in *Drosophila* [71] and showing the presence of the protein at LD/mitochondria contact sites in adipocytes [72] have significantly reinforced the hypothesis of a direct involvement of *C19orf12* in lipid metabolism and most probably in the transfer and/or utilization of lipids by the mitochondria. We can speculate that changes in intracellular lipid homeostasis can have detrimental consequences on other cellular processes, including mitochondrial respiration, autophagy flux, and iron handling, which are often perturbed in MPAN experimental models. Future studies will have to investigate the relevance and impact of these biochemical changes in neural cells and their connection with neural cell death, hopefully identifying molecular targets amenable to drug discovery.

## Figures and Tables

**Table 1:** Genes linked with NBIA disorders and corresponding functions.

Gene	NBIA Type	Protein Function
*Pantothenate kinase 2* (*PANK2*)	Pantothenate Kinase-Associated Neurodegeneration (PKAN)	Coenzyme A biosynthesis
*WD repeat domain 45* (*WDR45*)	β-propeller protein-associated neurodegeneration (BPAN)	Autophagy
*Phospholipase A2 group IV* (*PLA2G6*)	PLA2G6-Associated Neurodegeneration (PLAN)	Hydrolysis of membrane phospholipids
*C19orf12*	Mitochondrial Membrane Protein-associated Neurodegeneration (MPAN)	Lipid metabolism?
*Coenzyme A Synthase* (*COASY*)	COASY Protein-Associated Neurodegeneration (CoPAN)	Coenzyme A biosynthesis
*Ceruloplasmin* (*CP*)	Aceruloplasminemia	Oxidation of Fe^2+^ to Fe^3+^; trafficking of iron
*Ferritin light chain* (*FTL*)	Neuroferritinopathy	Iron storage
*Fatty acid 2-hydroxylase* (*FA2H*)	Fatty Acid Hydroxylase-associated Neurodegeneration (FAHN)	Generation of 2-hydroxylated phospholipids
*ATPase cation transporting 13A2* (*ATP13A2*)	Kufor–Rakeb Syndrome (KRS)	Translocation of ions and polyamines across the lysosomal membrane
*DDB1- and CUL4-associated factor 17* (*DCAF17*)	Woodhouse–Sakati Syndrome (WSS)	Protein ubiquitination
Other genes potentially involved in NBIA: *Sterol Carrier Protein 2* (*SCP2*), *Carnitine O-acetyltransferase* (*CRAT*), *Adaptor-related Protein complex-4*, *M1 Subunit* (*AP4M1*), *RALBP1-Associated Eps Domain Containing 1* (*REPS1*), *GTP Binding Protein 2* (*GTPBP2*), *Ferritin Heavy Chain 1* (*FTH1*).		

**Table 2:** Most relevant clinical features of MPAN.

Category	Common Clinical Features
Prevalence and genetics	Ultra-rare disease (approx. 1 in 1,000,000); autosomal recessive (AR-MPAN) or dominant (AD-MPAN) forms; biallelic or heterozygous mutations in *C19orf12*.
Age of onset	Typically, between 3 and 16 years (juvenile form), but can also occur in early adulthood.
Initial symptoms	Gait disturbances Lower limb spasticity (toe walking, spastic gait, frequent falls) Psychiatric abnormalities (behavioral changes, anxiety, inattention, hyperactivity) Vision impairment due to optic atrophy
Movement disorders	Spasticity Dystonia (localized or generalized) Parkinsonism (bradykinesia, rigidity, tremor) Dysarthria, dysphagia Amyotrophy, fasciculations due to motor axonal neuropathy, visible on electroneuromyogram
Psychiatric symptoms	Anxiety Inattention and hyperactivity Behavioral changes
Cognitive symptoms	Progressive cognitive decline Severe dementia in later stages
Visual disturbances	Optic atrophy Progressive vision loss
Multisystem involvement	Cardiac disease (cardiomyopathy, arrhythmias, and autonomic dysfunction), muscle weakness, bowel and bladder incontinence.
Advanced disease	Wheelchair dependence after 15 years of evolution Marked weight loss Death due to complications (e.g., aspiration pneumonia)
Brain MRI findings	Symmetric hypointensity in globus pallidus and substantia nigra on T2 sequences Internal medullary lamina (hyperintense band in globus pallidus) Possible eye of the tiger sign Iron accumulation in caudate, putamen, and cortical/cerebellar atrophy White matter hyperintensities in periventricular areas
Progression	Slow progression over 15 years, followed by plateau phase High variability in symptoms among patients

**Table 3:** List of the variants in the *C19orf12* gene linked with AR-MPAN described so far, with complete nomenclature information (numbers in parentheses are references).

AR-MPAN			
NM_001031726.3 (Isoform 1)		NM_031448.6 (Isoform 2)	
cDNA	Protein	cDNA	Protein
c.193+5G>A	p.? [37]	c.160+5G>A	p.?
c.194-2A>G	p.? [36,39]	c.161-2A>G	p.?
c.24G>C	p.Lys8Asn* [20]	c.-10G>C	p.?
c.32 C>T	p.Thr11Met [16,28,48,49]	c.-2C>T	p.?
c.52G>T	p.Asp18Tyr [49]	c.19G>T	p.Asp7Tyr
c.53A>G	p.Asp18Gly [48]	c.20A>G	p.Asp7Gly
c.94del	p.Met32fs* [36]	c.61del	p.Met21fs*
c.116C>T	p.Ser39Phe [11,36]	c.83C>T	p.Ser28Phe
c.142G>C	p.Ala48Pro [11,36]	c.109G>T	p.Ala37Ser
c.151T>G	p.Phe51Val [21,22,50]	c.118T>G	p.Phe40Val
c.157G>A	p.Gly53Arg [28,36]	c.124G>A	p.Gly42Arg
c.161del	p.Gly54Valfs*19 [51]	c.128del	p.Gly43Valfs*19
c.161G>A	p.Gly54Glu [28]	c.128G>A	p.Gly43Asp
c.171_181del	p.Gly58Argfs*21 [36]	c.138_148del	p.Gly47Argfs*21
c.172G>A	p.Gly58Ser [42]	c.139G>A	p.Gly47Ser
c.179C>T	p.Pro60Leu [11,36,52]	c.146C>T	p.Pro49Leu
c.187G>C	p.Ala63Pro [19,53]	c.154G>C	p.Ala52Pro
c.194G>T	p.Gly65Val [11,36]	c.161G>T	p.Gly54Val
c.194G>A	p.Gly65Glu [20,28,36]	c.161G>A	p.Gly54Glu
c.194-2del	p.Gly66_Gly73del [39]	c.161-2del	p.Gly55_Gly62del
c.196G>T	p.Gly66Trp [31,54,55]	c.163G>T	p.Gly55Trp
c.197_199del	p.Gly66del [16,19,48]	c.164_166del	p.Gly55del
c.199del	p.Ala67Leufs*6 [31,36,56,57]	c.166del	p.Ala56Leufs*6
c.199dup	p.Ala67Glyfs*16 [58]	c.166dup	p.Ala56Glyfs*16
c.204_214del	p.Gly69Argfs*10 [16,26,28,37,48]	c.171_181del	p.Gly58Argfs*10
c.205G>A	p.Gly69Arg [28,36,51]	c.172G>A	p.Gly58Arg
c.210dup	p.Leu71Alafs10* [59]	c.177dup	p.Leu60Alafs10*
c.215T>G	p.Leu72* [60]	c.182T>G	p.Leu61*
c.248C>T	p.Pro83Leu [11,28,56]	c.215C>T	p.Pro72Leu
c.287A>C	p.Gln96Pro [42,56]	c.254A>C	p.Gln85Pro
c.294G>C	p.Arg98Ser [11]	c.261G>C	p.Arg87Ser
c.362T>A	p.Leu121Gln [61]	c.329T>A	p.Leu110Gln
c.376_388del	p.Glu126Serfs*8 [26]	c.343_355del	p.Glu115Serfs*8
c.395 T>A	p.Leu132Gln [48,49]	c.362T>A	p.Leu121Gln
c.400G>C	p.Ala134Pro [11,56]	c.367G>C	p.Ala123Pro
c.404T>G	p.Met135Arg [62]	c.371T>G	p.Met124Arg
c.404dup	p.Met135Ilefs*17 [58]	c.371dup	p.Met124Ilefs*17
c.405del	p.Met135Ilefs*3 [45]	c.372del	p.Met124Ilefs*3
c.416 A>G	p.Tyr139Cys [56]	c.383A>G	p.Tyr128Cys
c.424A>G	p.Lys142Glu [28,35]	c.391A>G	p.Lys131Glu
c.436dup	p.Ala146Glyfs*6 [56]	c.403dup	p.Ala135Glyfs*6

**Table 4:** List of the variants in the *C19orf12* gene linked with AD-MPAN described so far, with complete nomenclature information (numbers in parentheses are references).

AD-MPAN			
NM_001031726.3 (Isoform 1)		NM_031448.6 (Isoform 2)	
cDNA	Protein	cDNA	Protein
c.227_237del	p.Met76Thrfs*3 [36]	c.194_204del	p.Met65Thrfs*3
c.238C>T	p.Gln80* [36]	c.205C>T	p.Gln69*
c.244A>T	p.Lys82* [9,56]	c.211A>T	p.Lys71*
c.256C>T	p.Gln86* [36,44]	c.223C>T	p.Gln75*
c.265_266del	p.Met89Glyfs*12 [43]	c.232_233del	p.Met78Glyfs*12
c.268G>T	p.Glu90* [36]	c.235G>T	p.Glu79*
c.273_274insA	p.Pro92Thrfs*10 [47]	c.240_241insA	p.Pro81Thrfs*10
c.278del	p.Pro93Leufs*26 [36,45]	c.245del	p.Pro82Leufs*26
c.278dup	p.Ala94Cysfs*8 [36]	c.245dup	p.Ala83Cysfs*8
c.279_282del	p.Ala94Serfs*24 [36]	c.246_249del	p.Ala83Serfs*24
c.279delT	p.Ala94Profs*25 [36]	c.246delT	p.Ala83Profs*25
c.283G > T	p.Glu95* [9]	c.250G>T	p.Glu84*
c.297insGCTC	p.Leu99fs*102 [42]	c.264insGCTC	p.Leu88fs*102
c.300del	p.Phe100Leufs*19 [36]	c.267del	p.Phe89Leufs*19
c.304G > T	p.Glu102* [9]	c.271G>T	p.Glu91*
c.335G>A	p.Trp112* [36,63]	c.302G>A	p.Trp101*
c.336_338delGACinsCACA	p.Trp112Cysfs*40 [46]	c.303_305delGACinsCACA	p.Trp101Cysfs*40
c.349C>T	p.Gln117* [36]	c.316C>T	p.GIn106*
c.357dup	p.Ala120Glyfs*32 [36]	c.324dup	p.Ala109Glyfs*32

## Supplementary Materials

The following supporting information can be downloaded at: https://www.mdpi.com/article/10.3390/brainsci15080777/s1, File S1: Materials and Methods.
