# Supplementary material for: An Update and Perspectives on Mitochondrial Membrane Protein-Associated Neurodegeneration and C19orf12 Research
Source: Brain Sci. 2025 Jul 22;15(8):777. doi: 10.3390/brainsci15080777 (PMC12384588; doi:10.3390/brainsci15080777)
Supplement: Supplementary file 1 [file brainsci-15-00777-s001.zip › brainsci-3657856-supplementary.pdf]

Materials and Methods

Transient transfection and immunofluorescence analysis

One day before transfection, cells were seeded at a density of 2 x 10^4 cells per imaging dish (Miltenyi Biotec, 130098284) in high glucose Dulbecco's Modified Eagle Medium without antibiotics. Transfection was performed using 1 µg DNA (C19orf12, NM_001031726.3, NM_031448.6, NM_001256046.3, NM_001282931.3 in pCMV6-AC-GFP) and Lipofectamine 2000 (Invitrogen) according to the manufacturer's protocol. HeLa cells were seeded at a density of 2x10^4 cells per imaging dish (Miltenyi Biotec, #130098284) in high glucose Dulbecco's Modified Eagle Medium without antibiotics. Transfection was performed using Lipofectamine™ 2000 Transfection Reagent (Invitrogen™, Waltham, MA, USA, #11668019) according to the manufacturer's protocol. 24 hours post-transfection, cells were fixed with 4% PFA. Primary antibodies (rabbit polyclonal anti-Calregulin, Santa Cruz, sc-11398; mouse monoclonal anti-ATP5A, Abcam, ab14748) and secondary antibodies (anti-rabbit IgG Alexa Fluor™ 647, anti-mouse IgG Alexa Fluor™ 568) were diluted in a 2% BSA, 0.1% NP-40 solution in PBS at 1:200 and 1:500, respectively. Cells were incubated with primary antibody O/N at 4°C, washed with 0.1% NP-40 solution in PBS, and then incubated with the appropriate secondary antibody for 1 hour at RT. Again, the cells were washed three times with PBS. The coverslips were then mounted with ProLong™ Gold Antifade Mountant, including DAPI (Fisher Scientific #P36931), and images were acquired by confocal microscopy (Leica TCS SP5).
